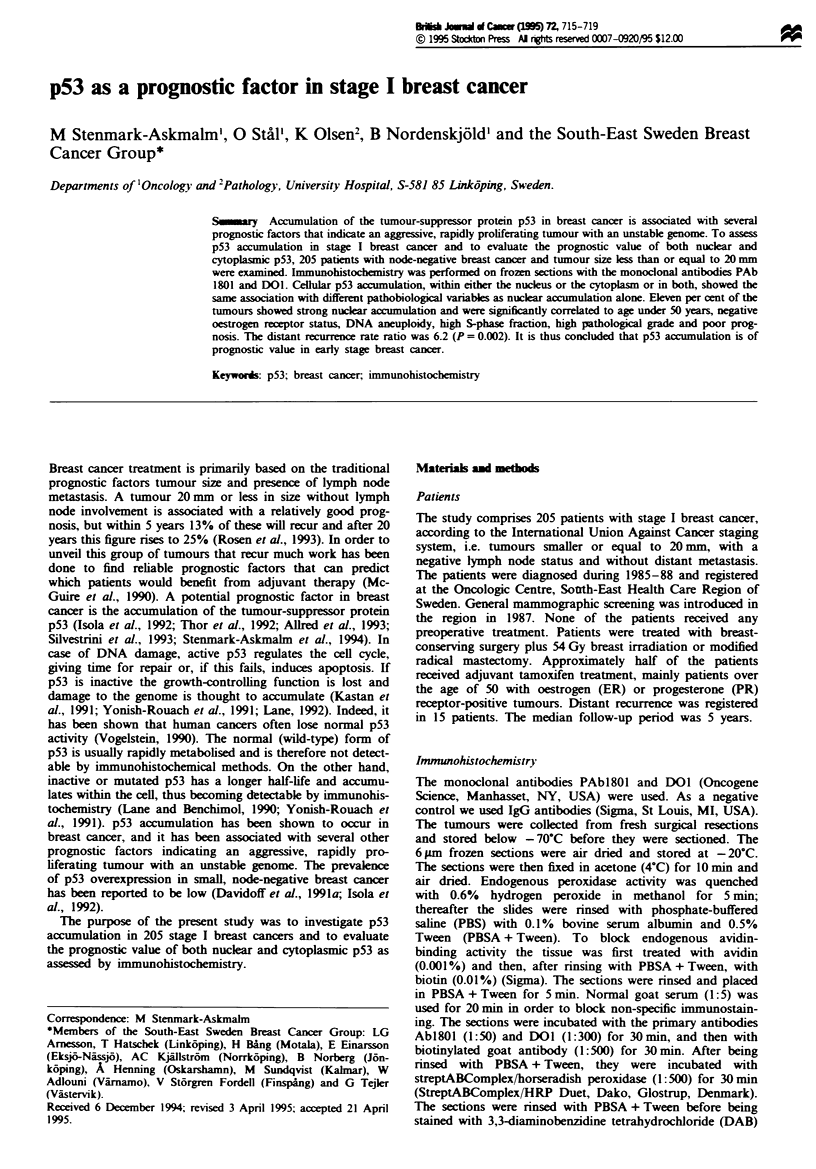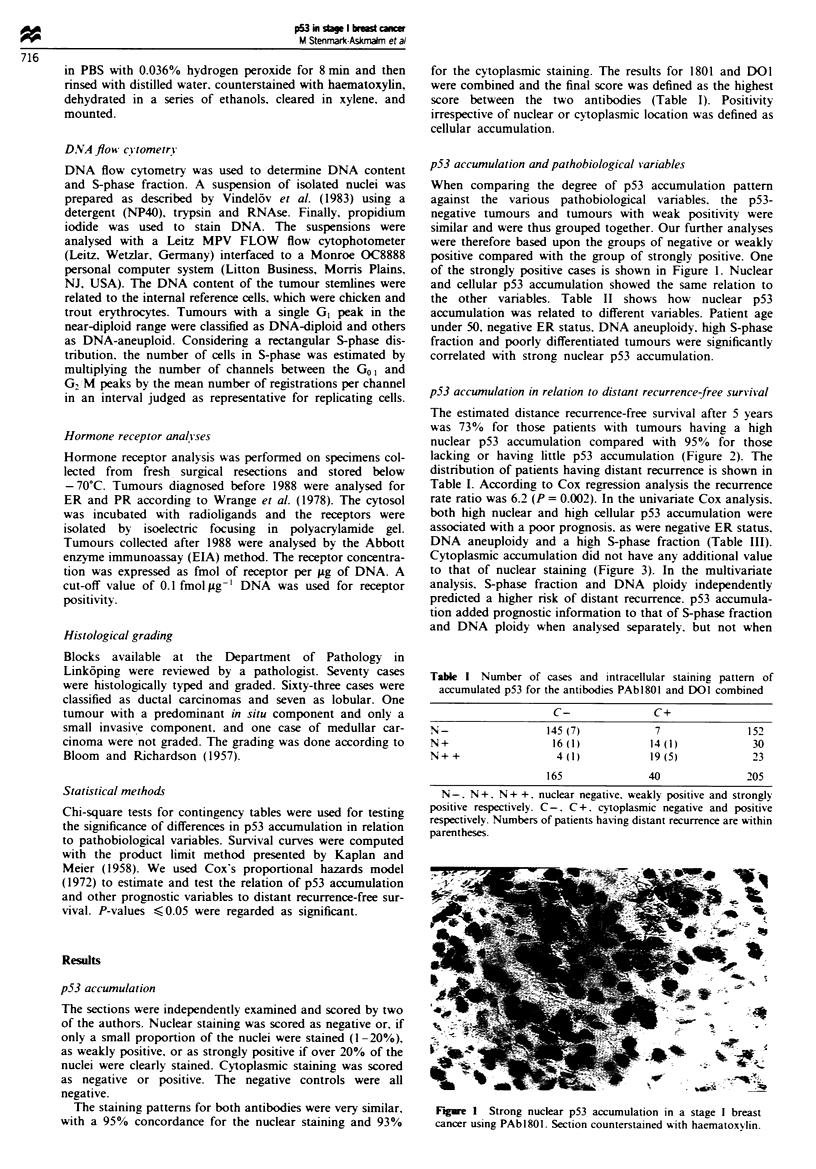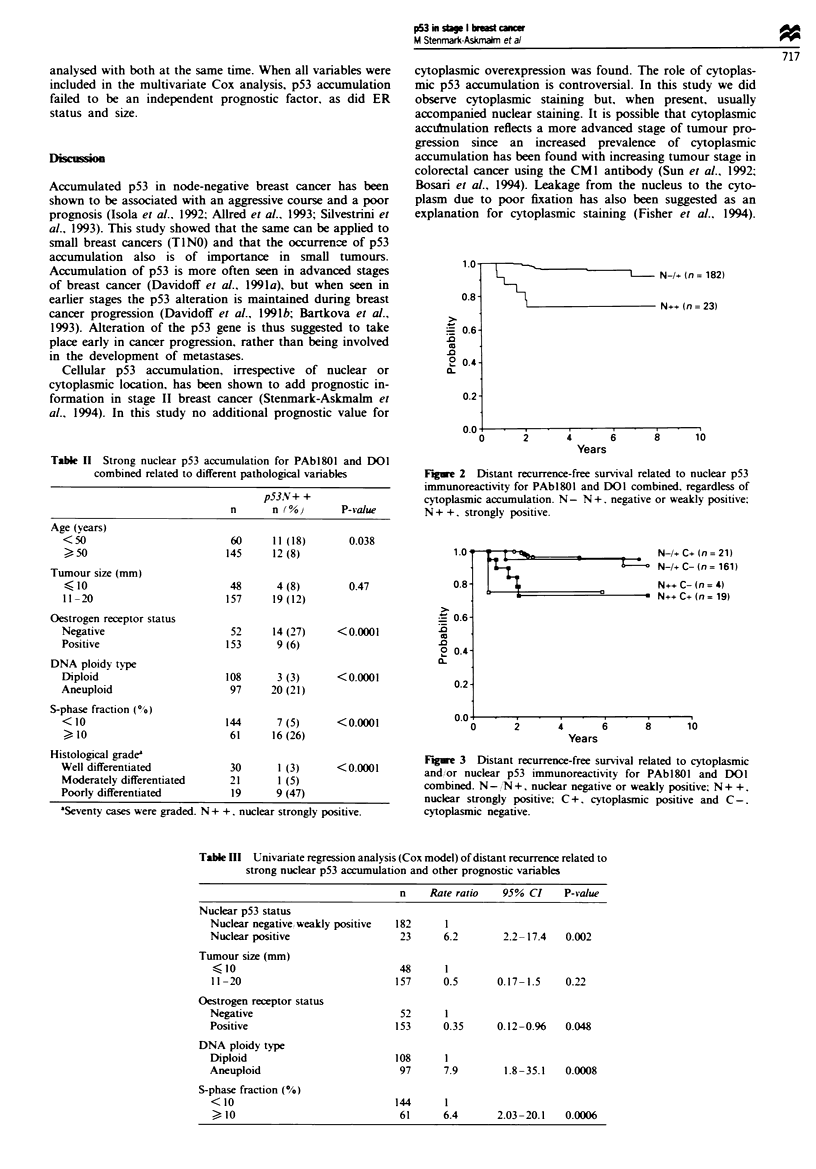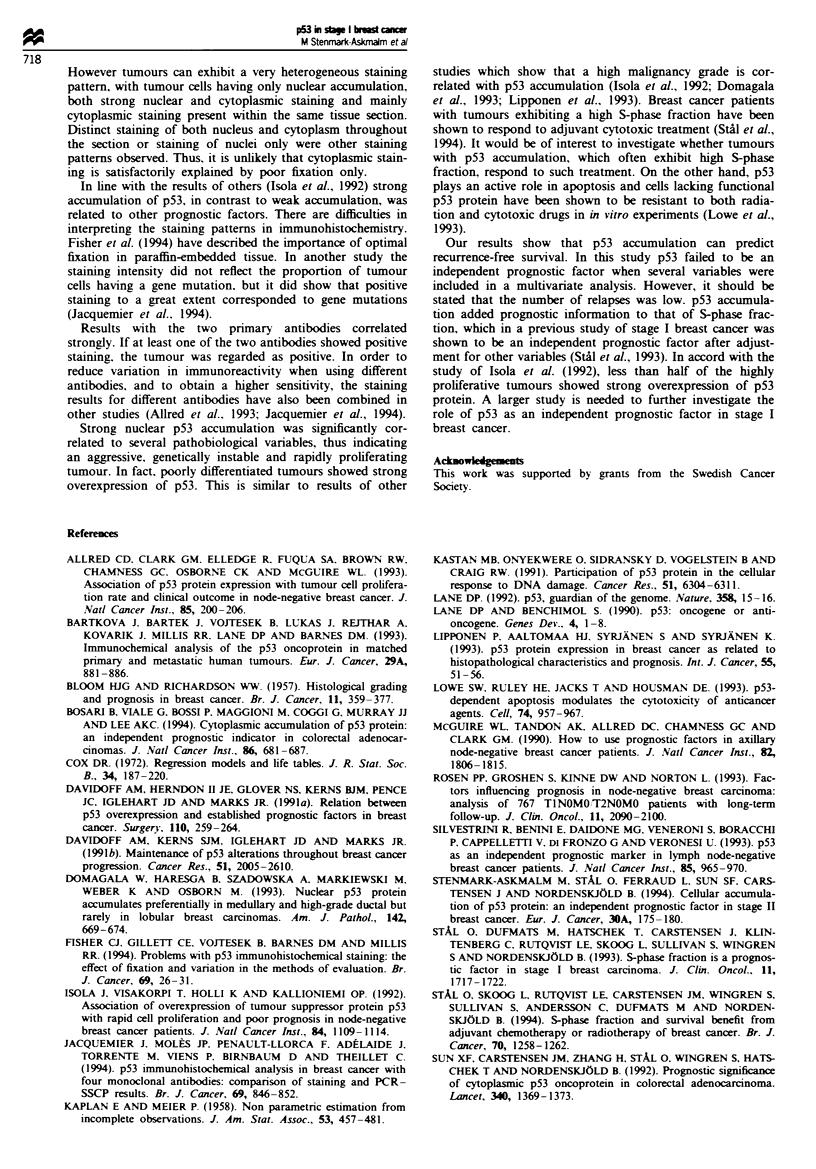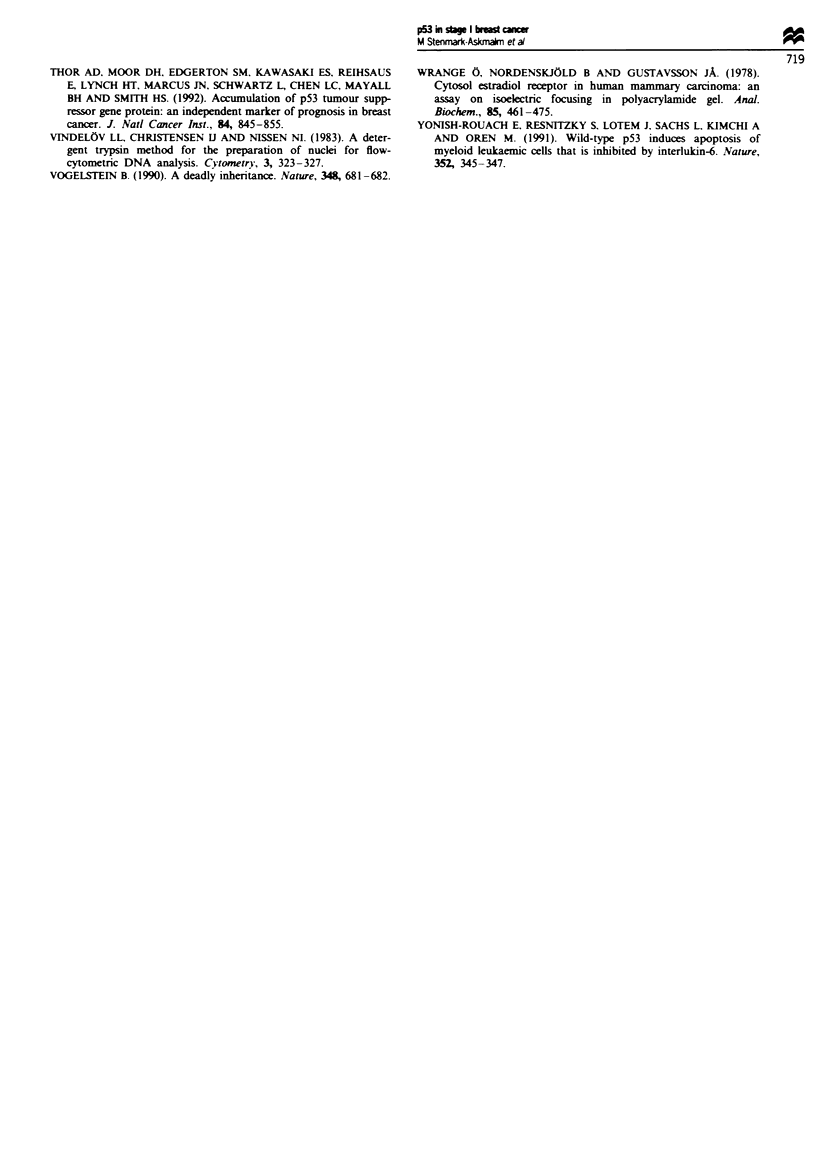# p53 as a prognostic factor in stage I breast cancer. South-East Sweden Breast Cancer Group.

**DOI:** 10.1038/bjc.1995.399

**Published:** 1995-09

**Authors:** M. Stenmark-Askmalm, O. Stål, K. Olsen, B. Nordenskjöld

**Affiliations:** Department of Oncology, University Hospital, Linköping, Sweden.

## Abstract

**Images:**


© 1995 Stockton Press All rights reserved 0007-0920/95 $12.00

### p53 as a prognostic factor in stage I breast cancer

M Stenmark-Askmalm[1], O Stål[1], K Olsen[2], B Nordenskjöld[1] and the South-East Sweden Breast Cancer Group*

*Departments of [1]Oncology and [2]Pathology, University Hospital, S-581 85 Linköping, Sweden.*

**Summary** Accumulation of the tumour-suppressor protein p53 in breast cancer is associated with several prognostic factors that indicate an aggressive, rapidly proliferating tumour with an unstable genome. To assess p53 accumulation in stage I breast cancer and to evaluate the prognostic value of both nuclear and cytoplasmic p53, 205 patients with node-negative breast cancer and tumour size less than or equal to 20 mm were examined. Immunohistochemistry was performed on frozen sections with the monoclonal antibodies PAb 1801 and DO1. Cellular p53 accumulation, within either the nucleus or the cytoplasm or in both, showed the same association with different pathobiological variables as nuclear accumulation alone. Eleven per cent of the tumours showed strong nuclear accumulation and were significantly correlated to age under 50 years, negative oestrogen receptor status, DNA aneuploidy, high S-phase fraction, high pathological grade and poor prognosis. The distant recurrence rate ratio was 6.2 ($P = 0.002$). It is thus concluded that p53 accumulation is of prognostic value in early stage breast cancer.

**Keywords**: p53; breast cancer; immunohistochemistry

Breast cancer treatment is primarily based on the traditional prognostic factors tumour size and presence of lymph node metastasis. A tumour 20 mm or less in size without lymph node involvement is associated with a relatively good prognosis, but within 5 years 13% of these will recur and after 20 years this figure rises to 25% (Rosen *et al.*, 1993). In order to unveil this group of tumours that recur much work has been done to find reliable prognostic factors that can predict which patients would benefit from adjuvant therapy (McGuire *et al.*, 1990). A potential prognostic factor in breast cancer is the accumulation of the tumour-suppressor protein p53 (Isola *et al.*, 1992; Thor *et al.*, 1992; Allred *et al.*, 1993; Silvestrini *et al.*, 1993; Stenmark-Askmalm *et al.*, 1994). In case of DNA damage, active p53 regulates the cell cycle, giving time for repair or, if this fails, induces apoptosis. If p53 is inactive the growth-controlling function is lost and damage to the genome is thought to accumulate (Kastan *et al.*, 1991; Yonish-Rouach *et al.*, 1991; Lane, 1992). Indeed, it has been shown that human cancers often lose normal p53 activity (Vogelstein, 1990). The normal (wild-type) form of p53 is usually rapidly metabolised and is therefore not detectable by immunohistochemical methods. On the other hand, inactive or mutated p53 has a longer half-life and accumulates within the cell, thus becoming detectable by immunohistochemistry (Lane and Benchimol, 1990; Yonish-Rouach *et al.*, 1991). p53 accumulation has been shown to occur in breast cancer, and it has been associated with several other prognostic factors indicating an aggressive, rapidly proliferating tumour with an unstable genome. The prevalence of p53 overexpression in small, node-negative breast cancer has been reported to be low (Davidoff *et al.*, 1991*a*; Isola *et al.*, 1992).

The purpose of the present study was to investigate p53 accumulation in 205 stage I breast cancers and to evaluate the prognostic value of both nuclear and cytoplasmic p53 as assessed by immunohistochemistry.

Correspondence: M Stenmark-Askmalm

*Members of the South-East Sweden Breast Cancer Group: LG Arnesson, T Hatschek (Linköping), H Bång (Motala), E Einarsson (Eksjö-Nässjö), AC Kjällström (Norrköping), B Norberg (Jönköping), Å Henning (Oskarshamn), M Sundqvist (Kalmar), W Adlouni (Värnamo), V Störgren Fordell (Finspång) and G Tejler (Västervik).

Received 6 December 1994; revised 3 April 1995; accepted 21 April 1995.

### Materials and methods

### *Patients*

The study comprises 205 patients with stage I breast cancer, according to the International Union Against Cancer staging system, i.e. tumours smaller or equal to 20 mm, with a negative lymph node status and without distant metastasis. The patients were diagnosed during 1985–88 and registered at the Oncologic Centre, South-East Health Care Region of Sweden. General mammographic screening was introduced in the region in 1987. None of the patients received any preoperative treatment. Patients were treated with breast-conserving surgery plus 54 Gy breast irradiation or modified radical mastectomy. Approximately half of the patients received adjuvant tamoxifen treatment, mainly patients over the age of 50 with oestrogen (ER) or progesterone (PR) receptor-positive tumours. Distant recurrence was registered in 15 patients. The median follow-up period was 5 years.

### *Immunohistochemistry*

The monoclonal antibodies PAb1801 and DO1 (Oncogene Science, Manhasset, NY, USA) were used. As a negative control we used IgG antibodies (Sigma, St Louis, MI, USA). The tumours were collected from fresh surgical resections and stored below −70°C before they were sectioned. The 6 μm frozen sections were air dried and stored at −20°C. The sections were then fixed in acetone (4°C) for 10 min and air dried. Endogenous peroxidase activity was quenched with 0.6% hydrogen peroxide in methanol for 5 min; thereafter the slides were rinsed with phosphate-buffered saline (PBS) with 0.1% bovine serum albumin and 0.5% Tween (PBSA + Tween). To block endogenous avidin-binding activity the tissue was first treated with avidin (0.001%) and then, after rinsing with PBSA + Tween, with biotin (0.01%) (Sigma). The sections were rinsed and placed in PBSA + Tween for 5 min. Normal goat serum (1:5) was used for 20 min in order to block non-specific immunostaining. The sections were incubated with the primary antibodies Ab1801 (1:50) and DO1 (1:300) for 30 min, and then with biotinylated goat antibody (1:500) for 30 min. After being rinsed with PBSA + Tween, they were incubated with streptABComplex/horseradish peroxidase (1:500) for 30 min (StreptABComplex/HRP Duet, Dako, Glostrup, Denmark). The sections were rinsed with PBSA + Tween before being stained with 3,3-diaminobenzidine tetrahydrochloride (DAB)

in PBS with 0.036% hydrogen peroxide for 8 min and then rinsed with distilled water, counterstained with haematoxylin, dehydrated in a series of ethanols, cleared in xylene, and mounted.

### *DNA flow cytometry*

DNA flow cytometry was used to determine DNA content and S-phase fraction. A suspension of isolated nuclei was prepared as described by Vindelöv *et al.* (1983) using a detergent (NP40), trypsin and RNAse. Finally, propidium iodide was used to stain DNA. The suspensions were analysed with a Leitz MPV FLOW flow cytophotometer (Leitz, Wetzlar, Germany) interfaced to a Monroe OC8888 personal computer system (Litton Business, Morris Plains, NJ, USA). The DNA content of the tumour stemlines were related to the internal reference cells, which were chicken and trout erythrocytes. Tumours with a single $G_1$ peak in the near-diploid range were classified as DNA-diploid and others as DNA-aneuploid. Considering a rectangular S-phase distribution, the number of cells in S-phase was estimated by multiplying the number of channels between the $G_{0/1}$ and $G_2/M$ peaks by the mean number of registrations per channel in an interval judged as representative for replicating cells.

### *Hormone receptor analyses*

Hormone receptor analysis was performed on specimens collected from fresh surgical resections and stored below −70°C. Tumours diagnosed before 1988 were analysed for ER and PR according to Wrange *et al.* (1978). The cytosol was incubated with radioligands and the receptors were isolated by isoelectric focusing in polyacrylamide gel. Tumours collected after 1988 were analysed by the Abbott enzyme immunoassay (EIA) method. The receptor concentration was expressed as fmol of receptor per μg of DNA. A cut-off value of 0.1 fmol $\mu g^{-1}$ DNA was used for receptor positivity.

### *Histological grading*

Blocks available at the Department of Pathology in Linköping were reviewed by a pathologist. Seventy cases were histologically typed and graded. Sixty-three cases were classified as ductal carcinomas and seven as lobular. One tumour with a predominant *in situ* component and only a small invasive component, and one case of medullar carcinoma were not graded. The grading was done according to Bloom and Richardson (1957).

### *Statistical methods*

Chi-square tests for contingency tables were used for testing the significance of differences in p53 accumulation in relation to pathobiological variables. Survival curves were computed with the product limit method presented by Kaplan and Meier (1958). We used Cox's proportional hazards model (1972) to estimate and test the relation of p53 accumulation and other prognostic variables to distant recurrence-free survival. *P*-values $\leqslant 0.05$ were regarded as significant.

### Results

### *p53 accumulation*

The sections were independently examined and scored by two of the authors. Nuclear staining was scored as negative or, if only a small proportion of the nuclei were stained (1–20%), as weakly positive, or as strongly positive if over 20% of the nuclei were clearly stained. Cytoplasmic staining was scored as negative or positive. The negative controls were all negative.

The staining patterns for both antibodies were very similar, with a 95% concordance for the nuclear staining and 93% for the cytoplasmic staining. The results for 1801 and DO1 were combined and the final score was defined as the highest score between the two antibodies (Table I). Positivity irrespective of nuclear or cytoplasmic location was defined as cellular accumulation.

### *p53 accumulation and pathobiological variables*

When comparing the degree of p53 accumulation pattern against the various pathobiological variables, the p53-negative tumours and tumours with weak positivity were similar and were thus grouped together. Our further analyses were therefore based upon the groups of negative or weakly positive compared with the group of strongly positive. One of the strongly positive cases is shown in Figure 1. Nuclear and cellular p53 accumulation showed the same relation to the other variables. Table II shows how nuclear p53 accumulation was related to different variables. Patient age under 50, negative ER status, DNA aneuploidy, high S-phase fraction and poorly differentiated tumours were significantly correlated with strong nuclear p53 accumulation.

### *p53 accumulation in relation to distant recurrence-free survival*

The estimated distance recurrence-free survival after 5 years was 73% for those patients with tumours having a high nuclear p53 accumulation compared with 95% for those lacking or having little p53 accumulation (Figure 2). The distribution of patients having distant recurrence is shown in Table I. According to Cox regression analysis the recurrence rate ratio was 6.2 ($P = 0.002$). In the univariate Cox analysis, both high nuclear and high cellular p53 accumulation were associated with a poor prognosis, as were negative ER status, DNA aneuploidy and a high S-phase fraction (Table III). Cytoplasmic accumulation did not have any additional value to that of nuclear staining (Figure 3). In the multivariate analysis, S-phase fraction and DNA ploidy independently predicted a higher risk of distant recurrence. p53 accumulation added prognostic information to that of S-phase fraction and DNA ploidy when analysed separately, but not when

**Table I** Number of cases and intracellular staining pattern of accumulated p53 for the antibodies PAb1801 and DO1 combined

| | *C−* | *C+* | |
|---|---|---|---|
| N− | 145 (7) | 7 | 152 |
| N+ | 16 (1) | 14 (1) | 30 |
| N++ | 4 (1) | 19 (5) | 23 |
| | 165 | 40 | 205 |

N−, N+, N++, nuclear negative, weakly positive and strongly positive respectively. C−, C+, cytoplasmic negative and positive respectively. Numbers of patients having distant recurrence are within parentheses.

**Figure 1** Strong nuclear p53 accumulation in a stage I breast cancer using PAb1801. Section counterstained with haematoxylin.

analysed with both at the same time. When all variables were included in the multivariate Cox analysis, p53 accumulation failed to be an independent prognostic factor, as did ER status and size.

### Discussion

Accumulated p53 in node-negative breast cancer has been shown to be associated with an aggressive course and a poor prognosis (Isola *et al.*, 1992; Allred *et al.*, 1993; Silvestrini *et al.*, 1993). This study showed that the same can be applied to small breast cancers (T1N0) and that the occurrence of p53 accumulation also is of importance in small tumours. Accumulation of p53 is more often seen in advanced stages of breast cancer (Davidoff *et al.*, 1991*a*), but when seen in earlier stages the p53 alteration is maintained during breast cancer progression (Davidoff *et al.*, 1991*b*; Bartkova *et al.*, 1993). Alteration of the p53 gene is thus suggested to take place early in cancer progression, rather than being involved in the development of metastases.

Cellular p53 accumulation, irrespective of nuclear or cytoplasmic location, has been shown to add prognostic information in stage II breast cancer (Stenmark-Askmalm *et al.*, 1994). In this study no additional prognostic value for cytoplasmic overexpression was found. The role of cytoplasmic p53 accumulation is controversial. In this study we did observe cytoplasmic staining but, when present, usually accompanied nuclear staining. It is possible that cytoplasmic accumulation reflects a more advanced stage of tumour progression since an increased prevalence of cytoplasmic accumulation has been found with increasing tumour stage in colorectal cancer using the CM1 antibody (Sun *et al.*, 1992; Bosari *et al.*, 1994). Leakage from the nucleus to the cytoplasm due to poor fixation has also been suggested as an explanation for cytoplasmic staining (Fisher *et al.*, 1994).

**Table II** Strong nuclear p53 accumulation for PAb1801 and DO1 combined related to different pathological variables

| | n | *p53N++* n (%) | P-*value* |
|---|---|---|---|
| Age (years) | | | |
| <50 | 60 | 11 (18) | 0.038 |
| ⩾50 | 145 | 12 (8) | |
| Tumour size (mm) | | | |
| ⩽10 | 48 | 4 (8) | 0.47 |
| 11-20 | 157 | 19 (12) | |
| Oestrogen receptor status | | | |
| Negative | 52 | 14 (27) | <0.0001 |
| Positive | 153 | 9 (6) | |
| DNA ploidy type | | | |
| Diploid | 108 | 3 (3) | <0.0001 |
| Aneuploid | 97 | 20 (21) | |
| S-phase fraction (%) | | | |
| <10 | 144 | 7 (5) | <0.0001 |
| ⩾10 | 61 | 16 (26) | |
| Histological grade[a] | | | |
| Well differentiated | 30 | 1 (3) | <0.0001 |
| Moderately differentiated | 21 | 1 (5) | |
| Poorly differentiated | 19 | 9 (47) | |

[a]Seventy cases were graded. N++, nuclear strongly positive.

**Figure 2** Distant recurrence-free survival related to nuclear p53 immunoreactivity for PAb1801 and DO1 combined, regardless of cytoplasmic accumulation. N− N+, negative or weakly positive; N++, strongly positive.

**Figure 3** Distant recurrence-free survival related to cytoplasmic and/or nuclear p53 immunoreactivity for PAb1801 and DO1 combined. N−/N+, nuclear negative or weakly positive; N++, nuclear strongly positive; C+, cytoplasmic positive and C−, cytoplasmic negative.

**Table III** Univariate regression analysis (Cox model) of distant recurrence related to strong nuclear p53 accumulation and other prognostic variables

| | n | *Rate ratio* | *95% CI* | P-*value* |
|---|---|---|---|---|
| Nuclear p53 status | | | | |
| Nuclear negative/weakly positive | 182 | 1 | | |
| Nuclear positive | 23 | 6.2 | 2.2-17.4 | 0.002 |
| Tumour size (mm) | | | | |
| ⩽10 | 48 | 1 | | |
| 11-20 | 157 | 0.5 | 0.17-1.5 | 0.22 |
| Oestrogen receptor status | | | | |
| Negative | 52 | 1 | | |
| Positive | 153 | 0.35 | 0.12-0.96 | 0.048 |
| DNA ploidy type | | | | |
| Diploid | 108 | 1 | | |
| Aneuploid | 97 | 7.9 | 1.8-35.1 | 0.0008 |
| S-phase fraction (%) | | | | |
| <10 | 144 | 1 | | |
| ⩾10 | 61 | 6.4 | 2.03-20.1 | 0.0006 |

However tumours can exhibit a very heterogeneous staining pattern, with tumour cells having only nuclear accumulation, both strong nuclear and cytoplasmic staining and mainly cytoplasmic staining present within the same tissue section. Distinct staining of both nucleus and cytoplasm throughout the section or staining of nuclei only were other staining patterns observed. Thus, it is unlikely that cytoplasmic staining is satisfactorily explained by poor fixation only.

In line with the results of others (Isola *et al.*, 1992) strong accumulation of p53, in contrast to weak accumulation, was related to other prognostic factors. There are difficulties in interpreting the staining patterns in immunohistochemistry. Fisher *et al.* (1994) have described the importance of optimal fixation in paraffin-embedded tissue. In another study the staining intensity did not reflect the proportion of tumour cells having a gene mutation, but it did show that positive staining to a great extent corresponded to gene mutations (Jacquemier *et al.*, 1994).

Results with the two primary antibodies correlated strongly. If at least one of the two antibodies showed positive staining, the tumour was regarded as positive. In order to reduce variation in immunoreactivity when using different antibodies, and to obtain a higher sensitivity, the staining results for different antibodies have also been combined in other studies (Allred *et al.*, 1993; Jacquemier *et al.*, 1994).

Strong nuclear p53 accumulation was significantly correlated to several pathobiological variables, thus indicating an aggressive, genetically instable and rapidly proliferating tumour. In fact, poorly differentiated tumours showed strong overexpression of p53. This is similar to results of other studies which show that a high malignancy grade is correlated with p53 accumulation (Isola *et al.*, 1992; Domagala *et al.*, 1993; Lipponen *et al.*, 1993). Breast cancer patients with tumours exhibiting a high S-phase fraction have been shown to respond to adjuvant cytotoxic treatment (Stål *et al.*, 1994). It would be of interest to investigate whether tumours with p53 accumulation, which often exhibit high S-phase fraction, respond to such treatment. On the other hand, p53 plays an active role in apoptosis and cells lacking functional p53 protein have been shown to be resistant to both radiation and cytotoxic drugs in *in vitro* experiments (Lowe *et al.*, 1993).

Our results show that p53 accumulation can predict recurrence-free survival. In this study p53 failed to be an independent prognostic factor when several variables were included in a multivariate analysis. However, it should be stated that the number of relapses was low. p53 accumulation added prognostic information to that of S-phase fraction, which in a previous study of stage I breast cancer was shown to be an independent prognostic factor after adjustment for other variables (Stål *et al.*, 1993). In accord with the study of Isola *et al.* (1992), less than half of the highly proliferative tumours showed strong overexpression of p53 protein. A larger study is needed to further investigate the role of p53 as an independent prognostic factor in stage I breast cancer.

**Acknowledgements**

This work was supported by grants from the Swedish Cancer Society.

### References

ALLRED CD, CLARK GM, ELLEDGE R, FUQUA SA, BROWN RW, CHAMNESS GC, OSBORNE CK AND MCGUIRE WL. (1993). Association of p53 protein expression with tumour cell proliferation rate and clinical outcome in node-negative breast cancer. *J. Natl Cancer Inst.*, **85**, 200-206.

BARTKOVA J, BARTEK J, VOJTESEK B, LUKAS J, REJTHAR A, KOVARIK J, MILLIS RR, LANE DP AND BARNES DM. (1993). Immunochemical analysis of the p53 oncoprotein in matched primary and metastatic human tumours. *Eur. J. Cancer*, **29A**, 881-886.

BLOOM HJG AND RICHARDSON WW. (1957). Histological grading and prognosis in breast cancer. *Br. J. Cancer*, **11**, 359-377.

BOSARI B, VIALE G, BOSSI P, MAGGIONI M, COGGI G, MURRAY JJ AND LEE AKC. (1994). Cytoplasmic accumulation of p53 protein: an independent prognostic indicator in colorectal adenocarcinomas. *J. Natl Cancer Inst.*, **86**, 681-687.

COX DR. (1972). Regression models and life tables. *J. R. Stat. Soc. B.*, **34**, 187-220.

DAVIDOFF AM, HERNDON II JE, GLOVER NS, KERNS BJM, PENCE JC, IGLEHART JD AND MARKS JR. (1991*a*). Relation between p53 overexpression and established prognostic factors in breast cancer. *Surgery*, **110**, 259-264.

DAVIDOFF AM, KERNS SJM, IGLEHART JD AND MARKS JR. (1991*b*). Maintenance of p53 alterations throughout breast cancer progression. *Cancer Res.*, **51**, 2005-2610.

DOMAGALA W, HARESGA B, SZADOWSKA A, MARKIEWSKI M, WEBER K AND OSBORN M. (1993). Nuclear p53 protein accumulates preferentially in medullary and high-grade ductal but rarely in lobular breast carcinomas. *Am. J. Pathol.*, **142**, 669-674.

FISHER CJ, GILLETT CE, VOJTESEK B, BARNES DM AND MILLIS RR. (1994). Problems with p53 immunohistochemical staining: the effect of fixation and variation in the methods of evaluation. *Br. J. Cancer*, **69**, 26-31.

ISOLA J, VISAKORPI T, HOLLI K AND KALLIONIEMI OP. (1992). Association of overexpression of tumour suppressor protein p53 with rapid cell proliferation and poor prognosis in node-negative breast cancer patients. *J. Natl Cancer Inst.*, **84**, 1109-1114.

JACQUEMIER J, MOLÈS JP, PENAULT-LLORCA F, ADÉLAIDE J, TORRENTE M, VIENS P, BIRNBAUM D AND THEILLET C. (1994). p53 immunohistochemical analysis in breast cancer with four monoclonal antibodies: comparison of staining and PCR-SSCP results. *Br. J. Cancer*, **69**, 846-852.

KAPLAN E AND MEIER P. (1958). Non parametric estimation from incomplete observations. *J. Am. Stat. Assoc.*, **53**, 457-481.

KASTAN MB, ONYEKWERE O, SIDRANSKY D, VOGELSTEIN B AND CRAIG RW. (1991). Participation of p53 protein in the cellular response to DNA damage. *Cancer Res.*, **51**, 6304-6311.

LANE DP. (1992). p53, guardian of the genome. *Nature*, **358**, 15-16.

LANE DP AND BENCHIMOL S. (1990). p53: oncogene or anti-oncogene. *Genes Dev.*, **4**, 1-8.

LIPPONEN P, AALTOMAA HJ, SYRJÄNEN S AND SYRJÄNEN K. (1993). p53 protein expression in breast cancer as related to histopathological characteristics and prognosis. *Int. J. Cancer*, **55**, 51-56.

LOWE SW, RULEY HE, JACKS T AND HOUSMAN DE. (1993). p53-dependent apoptosis modulates the cytotoxicity of anticancer agents. *Cell*, **74**, 957-967.

MCGUIRE WL, TANDON AK, ALLRED DC, CHAMNESS GC AND CLARK GM. (1990). How to use prognostic factors in axillary node-negative breast cancer patients. *J. Natl Cancer Inst.*, **82**, 1806-1815.

ROSEN PP, GROSHEN S, KINNE DW AND NORTON L. (1993). Factors influencing prognosis in node-negative breast carcinoma: analysis of 767 T1N0M0/T2N0M0 patients with long-term follow-up. *J. Clin. Oncol.*, **11**, 2090-2100.

SILVESTRINI R, BENINI E, DAIDONE MG, VENERONI S, BORACCHI P, CAPPELLETTI V, DI FRONZO G AND VERONESI U. (1993). p53 as an independent prognostic marker in lymph node-negative breast cancer patients. *J. Natl Cancer Inst.*, **85**, 965-970.

STENMARK-ASKMALM M, STÅL O, FERRAUD L, SUN SF, CARSTENSEN J AND NORDENSKJÖLD B. (1994). Cellular accumulation of p53 protein: an independent prognostic factor in stage II breast cancer. *Eur. J. Cancer*, **30A**, 175-180.

STÅL O, DUFMATS M, HATSCHEK T, CARSTENSEN J, KLINTENBERG C, RUTQVIST LE, SKOOG L, SULLIVAN S, WINGREN S AND NORDENSKJÖLD B. (1993). S-phase fraction is a prognostic factor in stage I breast carcinoma. *J. Clin. Oncol.*, **11**, 1717-1722.

STÅL O, SKOOG L, RUTQVIST LE, CARSTENSEN JM, WINGREN S, SULLIVAN S, ANDERSSON C, DUFMATS M AND NORDENSKJÖLD B. (1994). S-phase fraction and survival benefit from adjuvant chemotherapy or radiotherapy of breast cancer. *Br. J. Cancer*, **70**, 1258-1262.

SUN XF, CARSTENSEN JM, ZHANG H, STÅL O, WINGREN S, HATSCHEK T AND NORDENSKJÖLD B. (1992). Prognostic significance of cytoplasmic p53 oncoprotein in colorectal adenocarcinoma. *Lancet*, **340**, 1369-1373.

THOR AD, MOOR DH, EDGERTON SM, KAWASAKI ES, REIHSAUS E, LYNCH HT, MARCUS JN, SCHWARTZ L, CHEN LC, MAYALL BH AND SMITH HS. (1992). Accumulation of p53 tumour suppressor gene protein: an independent marker of prognosis in breast cancer. *J. Natl Cancer Inst.*, **84**, 845-855.

VINDELÖV LL, CHRISTENSEN IJ AND NISSEN NI. (1983). A detergent trypsin method for the preparation of nuclei for flowcytometric DNA analysis. *Cytometry*, **3**, 323-327.

VOGELSTEIN B. (1990). A deadly inheritance. *Nature*, **348**, 681-682.

WRANGE Ö, NORDENSKJÖLD B AND GUSTAVSSON JÅ. (1978). Cytosol estradiol receptor in human mammary carcinoma: an assay on isoelectric focusing in polyacrylamide gel. *Anal. Biochem.*, **85**, 461-475.

YONISH-ROUACH E, RESNITZKY S, LOTEM J, SACHS L, KIMCHI A AND OREN M. (1991). Wild-type p53 induces apoptosis of myeloid leukaemic cells that is inhibited by interlukin-6. *Nature*, **352**, 345-347.